# Infliximab-Induced Autoimmune Phenomenon in an Adolescent With Ulcerative Colitis: Drug-Induced Lupus vs Inflammatory Myositis

**DOI:** 10.7759/cureus.97112

**Published:** 2025-11-17

**Authors:** Nika Bakshi, Maya S Kardouh, Jamal Kriem

**Affiliations:** 1 Medicine, Oakland University William Beaumont School of Medicine, Rochester, USA; 2 Internal Medicine, Oakland University William Beaumont School of Medicine, Rochester, USA; 3 Pediatric Gastroenterology, Beaumont Health, Royal Oak, USA

**Keywords:** autoimmune adverse events, inflammatory bowel disease, infliximab, myositis, ulcerative colitis

## Abstract

We present the case of a 15-year-old female with ulcerative colitis who developed an asymptomatic rise in creatine kinase and transaminases while on infliximab therapy. Additional rheumatologic workup revealed high-titer anti-nuclear antibody and positive anti-histone antibody, with the absence of myositis-specific autoantibodies. A drug-induced autoimmune phenomenon was suspected, with the patient lacking any muscle weakness or lupus features. The patient was subsequently switched to ustekinumab, which resulted in complete normalization of her labs while maintaining remission of her ulcerative colitis.

## Introduction

Ulcerative colitis (UC) is a chronic inflammatory bowel disease of the large intestine that is limited to the mucosal layers of the colon [1]. Patients are often treated with a range of pharmacologic therapies, ranging from biologic agents, oral sphingosine 1-phosphate receptor modulators, or glucocorticoids [1]. Infliximab, a tumor necrosis factor-alpha (TNF-ɑ) inhibitor, is a highly effective and widely used biologic agent in the treatment of moderate-to-severe active pediatric UC [2]. 

Despite its success in achieving clinical remission, infliximab carries the risk of autoimmune adverse events, ranging from drug-induced lupus to drug-induced inflammatory myositis [3]. It has been established that TNF-ɑ inhibitors can induce this autoimmunity by immune dysregulation, such as the production of autoantibodies [4]. Among these complications, drug-induced lupus is a more commonly recognized phenomenon, while infliximab-induced inflammatory myositis is exceedingly rare [3,4]. 

Currently, only a few cases of infliximab-induced inflammatory myositis have been described in the literature, and, to our knowledge, no cases have been reported in pediatric patients [3,5-7]. There have only been a few reports of infliximab-induced inflammatory myositis in adults, such as facial myositis and diffuse myositis in a patient with sarcoidosis and lupus pernio [3,5]. Adult cases of drug-induced myositis have also been attributed to a combination of infliximab and azathioprine [6,7]. 

In this case report, we describe a case of an adolescent patient with UC who developed asymptomatic elevations in creatine kinase (CK) and transaminases, along with positive anti-nuclear antibody (ANA) and positive anti-histone antibodies while on infliximab. Although diagnostic tests, such as a muscle biopsy, were not performed due to improving laboratory results and lack of clinical symptoms, the overall presentation raised concerns for both an infliximab-induced myositis and drug-induced lupus. We highlight the diagnostic uncertainty between infliximab-induced inflammatory myositis and drug-induced lupus and the importance of close laboratory monitoring, especially in detecting asymptomatic autoimmune reactions to infliximab.

## Case presentation

A 13-year-old female patient was diagnosed with UC when a colonoscopy with biopsy revealed ulcerative pancolitis. The patient was started on infliximab 10 mg/kg shortly after her diagnosis, starting at 10 mg/kg every eight weeks and eventually increasing to every six weeks due to persistent abdominal pain and bloody stools. The patient achieved a good clinical response, maintaining remission since diagnosis. In addition, she was started on extended-release budesonide 9 mg daily due to active disease, which was later successfully titrated down to 3 mg every other day. Her medical history was significant for arthritis and iron-deficiency anemia. 

Throughout the past three months, the patient was developing a gradual increase in hepatic transaminases, with aspartate aminotransferase (AST) rising from 38 U/L to 44 U/L and eventually to 63 U/L, and alanine aminotransferase (ALT) rising from 43 U/L to 55 U/L and eventually to 84 U/L (Table 1). Given this upward trend, additional labs were ordered, including CK and gamma-glutamyl transferase (GGT). CK was elevated to 385 U/L (reference range: 30-150 U/L), while GGT was mildly elevated at 26 U/L (reference range: 7-21 U/L) (Table 1).

**Table 1:** Chronological trend of laboratory parameters Progressive elevation of AST and ALT prompted the evaluation of CK and GGT, followed by normalization after discontinuing infliximab and initiating ustekinumab. ALT, alanine aminotransferase; AST, aspartate aminotransferase; CK, creatine kinase; GGT, gamma-glutamyl transferase.

Timeline	Laboratory test	Result (U/L)	Reference range (U/L)	Comment
Initial routine monitoring (first noted elevation)	AST	38	<35	Mildly elevated
Initial routine monitoring (first noted elevation)	ALT	53	8-22	Mildly elevated
Three months later	AST	63	<35	Further increased
Three months later	ALT	84	8-22	Further increased
Three months later	GGT	26	7-21	Mildly elevated
Three months later	CK	385	30-150	Elevated
Eighty-eight days after discontinuing infliximab/55 days after the first ustekinumab dose	AST	22	<35	Improved
Eighty-eight days after discontinuing infliximab/55 days after the first ustekinumab dose	ALT	27	8-22	Improved
Six months after the initial CK evaluation	CK	169	30-150	Decreasing
Twelve months after the initial CK evaluation	CK	116	30-150	Normalized

Given the elevation in ALT and AST, a subsequent workup was performed, including autoimmune hepatitis, Wilson disease, thyroid-stimulating hormone, viral serologies, inflammatory markers, celiac screening, alpha-1 antitrypsin deficiency, and infectious hepatitis. Screening demonstrated ANA titers of 1:1280, negative hepatitis A, B, and C, negative liver kidney microsomal antibodies and anti-smooth muscle antibodies, and a CK of 385 U/L (reference range: 30-150 U/L). The combination of transaminitis, high-titer ANA, elevated CK levels, and negative screening for other etiologies prompted a rheumatology referral for further evaluation. An autoimmune and myositis-specific workup was performed and was positive again for ANA and anti-histone antibody (Table 2). Of note, aldolase was slightly elevated at 8 IU/L (reference range: 1-7 IU/L).

**Table 2:** Autoimmune and myositis-specific workup Positive ANA and positive anti-histone antibodies in the setting of negative anti-dsDNA, ENA, and myositis-specific antibodies. These findings were consistent with an infliximab-associated autoimmune laboratory abnormality. Of note, the discrepancy between a positive ANA titer and a negative ANA screen reflects varying testing methodologies. An ANA screen is performed by a multiplex immunoassay, while the ANA titer is determined by a more sensitive, indirect immunofluorescence assay. ANA, anti-nuclear antibody; dsDNA Ab, double-stranded deoxyribonucleic acid antibody.

Test	Result	Reference range/interpretation
Anti-dsDNA Ab	<9.8 IU/mL	<27 IU/mL = negative; 27-35 IU/mL = borderline positive; ≥36 IU/mL = positive
ANA screen	Negative	Negative = within normal limits
ANA titer	≥1:1280	Titer
Histone Ab	1.3	<1 = negative; 1.0-1.5 = weak positive; 1.6-2.5 = moderate positive >2.5 = strong positive
Myomarker 3 panel–qualitative antibodies	All negative (PL-7, PL-12, EJ, OJ, Mi-2, SRP, Ku, U2 RNP, and U3 RNP)	Negative = within normal limits
Myomarker 3 panel–quantitative antibodies	All <20 U (Jo-1, TIF-1y, MDA-5, NXP-2, PM/Scl-100, SS-A, and U1 RNP)	<20 U = negative
ANA screen (multiplex bead/extractable nuclear antigen (ENA) panel)	All negative (anti-RNP, anti-Scl-70, anti-SmRN, anti-SSa/anti-Ro, anti-SSb/anti-La, ANA screen, chromatin, and ribosomal protein)	Negative = within normal limits

At the time of this workup, the patient reported feeling well, denying any skin rashes, musculoskeletal pain, stiffness, or weakness. In addition to the patient being asymptomatic, her physical exam was unremarkable, with 5/5 strength throughout, normal deep tendon reflexes, and a lack of myalgia, skin rashes, or lesions. 

As the new-onset transaminitis, positive ANA titer, positive anti-histone antibody, elevated CK levels, and slightly elevated aldolase coincided with the initiation of infliximab, an infliximab-induced autoimmune phenomenon was suspected. Muscle biopsy and imaging were deferred since the patient lacked clinical symptoms and had improvement after medication changes. 

The patient was switched from infliximab to ustekinumab, an inhibitor of interleukin-12 (IL-12) and IL-23 [8]. She started on a one-time intravenous dose of 260 mg ustekinumab followed by 90 mg subcutaneously every 8 weeks. Approximately a month later, ustekinumab was adjusted to 90 mg subcutaneously every 4 weeks due to abdominal pain and bloody stools. Due to difficulties obtaining insurance coverage on 90 mg ustekinumab subcutaneously every 4 weeks, the patient was switched to 130 mg ustekinumab intravenously every 4 weeks instead. 

Despite increasing the dose of ustekinumab, the patient continued to experience inadequate relief of symptoms, such as persistent abdominal pain and a month of bloody stools. In order to achieve adequate control of her symptoms, the patient was switched to a Janus kinase inhibitor, upadacitinib, 45 mg daily for two months, followed by a dose of 15 mg daily. 

Eighty-eight days after discontinuing infliximab and fifty-five days after receiving the first dose of IV ustekinumab, the patient had significant improvement in her AST from 63 U/L to 22 U/L (reference range: <35 U/L) and ALT from 84 U/L to 27 U/L (reference range: 8-22 U/L), as summarized in Table 1. At a subsequent follow-up visit, approximately six months later, her CK levels had decreased from 385 U/L to 169 U/L (reference range: 30-150 U/L). Twelve months after that visit, the CK levels normalized to 116 U/L.

Clinically, the patient reported an improvement in her symptoms following the switch to upadacitinib. During follow-ups, she denied any abdominal pain, nausea, or vomiting. There has been no recurrence of myositis or transaminitis during follow-ups. 

## Discussion

The setting of new-onset transaminitis and positive autoantibodies in a patient on infliximab therapy prompted a broad differential diagnosis. This differential diagnosis included idiopathic inflammatory myopathies, such as juvenile dermatomyositis (JDM), benign hyperCKemia, drug-induced lupus erythematosus (DILE), and drug-induced inflammatory myositis. 

Idiopathic inflammatory myopathy, particularly JDM, was deemed less likely given the lack of clinical findings consistent with JDM, such as a characteristic heliotrope rash or Gottron’s papules [8]. Furthermore, the patient lacked any muscle weakness or capillaroscopy changes and had negative myositis-specific antibodies as observed in Table 1 [8]. Benign hyperCKemia was also unlikely since the positive ANA and anti-histone antibodies suggested an immune-mediated mechanism. 

We also suspected DILE, since the patient had high-titer ANA and weakly positive anti-histone antibodies. It is important to note that the patient lacked typical cutaneous or systemic manifestations of lupus, such as rash, serositis, arthritis, fever, myalgia, or malaise [9]. As highlighted in the literature, there are no definitive criteria or diagnostic tests for the diagnosis of DILE [9]. However, the following guidelines have been proposed: (a) exposure to a drug associated with DILE for at least one month, (b) development of at least one characteristic clinical feature of lupus, (c) a positive ANA, and (d) resolution of clinical findings after medication discontinuation [9]. In the case of our patient, the lack of clinical symptoms made it difficult to confidently confirm a diagnosis of DILE. 

We also suspected a rare but reported adverse reaction to infliximab, infliximab-induced inflammatory myositis. The patient was on a known offending agent, a TNF-ɑ inhibitor, which has been associated with the development of autoantibodies and causing drug-induced inflammatory myositis [6,7]. This has been confirmed in a few case reports focusing on adults, with most published case reports confirming the diagnosis through muscle biopsy [7]. Ultimately, diagnostic tests such as a muscle biopsy were not performed due to the resolution of laboratory abnormalities after discontinuation of infliximab and the lack of clinical symptoms. 

In summary, this case emphasizes the complexity in diagnosing drug-induced autoimmune conditions in patients who are asymptomatic with laboratory abnormalities. In the case of our patient, the absence of symptoms made it difficult to definitively confirm a diagnosis of DILE. We also deferred diagnostic testing for myositis since the patient was asymptomatic, contributing to uncertainty in distinguishing between DILE and infliximab-induced inflammatory myositis. Regardless, these findings demonstrate the need for individualized decision-making in stable patients with serologic changes, especially those on biologic therapy. 

## Conclusions

This case highlights the importance of regular laboratory monitoring in pediatric patients receiving infliximab, as early detection can ensure safe transition to alternative treatment. Even in the absence of symptoms, clinicians should remain cautious for drug-induced autoimmune phenomena in patients on TNF-ɑ inhibitors. 

## References

[1] Cohen RD, Stein AC (2025). UpToDate: Management of moderate to severe ulcerative colitis in adults. UpToDate.

[2] Puthoor PR, de Zoeten EF (2013). Pediatric ulcerative colitis: the therapeutic road to infliximab. Biol Ther.

[3] Mukharesh L, Tinsley A (2022). Infliximab-induced optic perineuritis and facial myositis. J Neuroophthalmol.

[4] Gerriets V, Goyal A, Khaddour K (2023). Tumor necrosis factor inhibitors. StatPearls [Internet].

[5] Zhou Y, Lower EE, Li H, Baughman RP (2016). Sarcoidosis patient with lupus pernio and infliximab-induced myositis: response to Acthar gel. Respir Med Case Rep.

[6] Masnammany AM, Lau W, Wong PK, Manolios N (2022). Anti-tumor necrosis factor-induced focal myositis. Proc Singapore Healthc.

[7] Yoshida A, Katsumata Y, Hirahara S, Hanaoka M, Ochiai M, Kobayashi M, Harigai M (2021). Tumour necrosis factor inhibitor-induced myositis in a patient with ulcerative colitis. Mod Rheumatol Case Rep.

[8] Gara S, Jamli RT, Muse ME, Litaeim N (2023). Juvenile dermatomyositis. StatPearls [Internet].

[9] Merola JF (2025). Drug-induced lupus. UpToDate.

